# Imaging assessment of surgical repair of knee cartilage

**DOI:** 10.1590/0100-3984.2020.53.4e3

**Published:** 2020

**Authors:** Marcelo R. de Abreu

**Affiliations:** 1 Hospital Mãe de Deus, Porto Alegre, RS, Brazil. Email: marcelorad@gmail.com.

Cartilage injuries are common, frequently affect young patients, and have the potential to progress to osteoarthritis. Because articular cartilage lacks vascularity, most chondral defects do not heal spontaneously and surgical repair may be required. A number of surgical techniques are available to treat focal chondral defects, including marrow stimulation, osteochondral autografting or allografting, and autologous chondrocyte implantation^(1,2)^.

Patients can benefit greatly from cartilage repair surgery, and there is need for a high-quality, noninvasive imaging modality to assess the structure and biochemical properties of the repair tissue. Although arthroscopy is considered the standard of reference for the evaluation of cartilage before and after repair, it is invasive, is associated with morbidity, and cannot adequately depict the deep cartilage layer or underlying bone. Magnetic resonance imaging (MRI) is currently the best imaging technique available for the assessment of repair joint tissue from the articular surface to the bone-cartilage interface. Imaging of repair cartilage is needed in order to determine the extent of defect filling, the degree of peripheral integration with the host tissue, the morphologic structure and signal intensity of the repair tissue, and the integrity of the host cartilage^(3,4)^. The normal postoperative appearance of the joints after cartilage repair varies according to the surgical technique used and the stage of healing.

Quantitative MRI techniques such as T1, T2, and T2* mapping^(5)^, as well as delayed gadolinium-enhanced MRI of cartilage, help assess collagen content/orientation, water content, glycosaminoglycan content, and proteoglycan content, not only in the repair tissue as it matures but also in the “native” cartilage^(1,2,5)^. When these techniques are employed in cases of cartilage repair follow-up, the following questions have to be answered: Do T2 relaxation times differ between repair tissues and adjacent native cartilage?; Are the differences reduced over time?; and Is there a difference between a global assessment and a line profile analysis? Automation and standardization of these novel sequences will improve the assessment of hyaline cartilage and cartilage repair procedures.

To identify potential complications, it is important to be familiar with the various repair procedures and the characteristic MRI features of the repair tissue at various postoperative intervals. The validation of cartilage repair techniques calls for short-, medium-, and long-term follow-up. Follow-up MRI studies should be performed at 3-6 months after the surgical procedure in order to assess the volume and integration of the repair tissue. Subsequent imaging in the first postoperative year allows the maturation of the graft to be evaluated and any complications to be identified. The timing of the follow-up evaluations continues to be a problem in cases of cartilage repair because of the slow progression of cartilage degeneration over time^(1)^.

In the previous issue of **Radiologia Brasileira**, Souza et al.^(6)^ provided a complete didactic pictorial essay of surgical repair of knee cartilage evaluated with MRI.
